# Arterial Stiffness in Breast Cancer Patients Treated with Anthracycline and Trastuzumab-Based Regimens

**DOI:** 10.1155/2018/5352914

**Published:** 2018-05-02

**Authors:** Özlem Yersal, Ufuk Eryilmaz, Hakan Akdam, Nezih Meydan, Sabri Barutca

**Affiliations:** ^1^Oncology Department, Internal Medicine Department, Adnan Menderes University, Aydin, Turkey; ^2^Nephrology Department, Internal Medicine Department, Adnan Menderes University, Aydin, Turkey; ^3^Cardiology Department, Adnan Menderes University, Aydin, Turkey

## Abstract

**Aims:**

Cardiovascular diseases are the primary cause of premature morbidity and mortality in early breast cancer patients after treatment with cardiotoxic chemotherapeutic agents. Arterial stiffness is an independent risk factor for future cardiovascular diseases and can be used as a predictive marker of subclinical cardiac damage. The aim of this study is to analyze the arterial stiffness in breast cancer patients who are in the follow-up period after receiving anthracycline-based chemotherapy regimens with trastuzumab.

**Methods and Material:**

We enrolled 45 HER2-positive breast cancer patients who are on follow-up at least for six months after completion of adjuvant chemotherapy with trastuzumab, and cardiovascular risk matched 30 control volunteers. The measurements were done with pulse wave analyzing machine.

**Results:**

Mean pulse wave velocity was higher in breast cancer patients compared to controls. The pulse wave velocity was significantly higher in patients receiving aromatase inhibitors compared to patients under tamoxifen. It was also significantly higher in postmenopausal breast cancer patients than postmenopausal controls.

**Conclusions:**

Arterial stiffness measurements may predict the breast cancer survivors with higher risk for cardiovascular events earlier in the follow-up period, and necessary preventive approaches and/or treatments can be applied.

## 1. Introduction

Anthracycline-based chemotherapy followed by a taxane and trastuzumab combination is the most commonly preferred regimen in high-risk HER2-positive breast cancer patients without cardiac dysfunction [1–5]. Cardiotoxicity is the major restricting factor for the use of trastuzumab. Trastuzumab cardiotoxicity is characterized by an asymptomatic decrease in left ventricular ejection fraction or clinical heart failure on a lesser extent. Anthracyclines also may cause acute and chronic cardiotoxicity. Chronic toxicity is associated with the cumulative dose of anthracycline and observed as cardiomyopathy in the clinical setting. In clinical trials, patients receiving adjuvant treatment with trastuzumab 5–10% left ventricular dysfunction and congestive heart failure have been reported. This rate increases up to 27% with the simultaneous use of anthracyclines and trastuzumab [6].

Cardiovascular diseases are the most common cause of premature morbidity and mortality in breast cancer survivors [7]. Anthracycline cumulative dose is restricted to reduce the risk of cardiovascular disease. However, lower doses of anthracyclines were shown to be associated with myocardial degenerative process demonstrated by endomyocardial biopsies prior to any functional abnormality [8, 9]. Sequential trastuzumab administration to anthracyclines also may enhance subclinical cardiovascular damage and therefore, there is need for indicators that may show early and potentially reversible cardiovascular dysfunction.

Arterial stiffness is an independent risk factor for future cardiovascular events, and it can be used as an indicator of subclinical cardiac damage. Arterial stiffness is involved in the pathophysiology of myocardial infarction, stroke, or heart failure especially in patients with hypertension and diabetes mellitus [10].

In this study, we aimed to evaluate arterial stiffness of breast cancer survivors who have been exposed to trastuzumab and anthracycline containing regimens.

## 2. Methods

### 2.1. Patient Selection

This study was approved by the institutional review board; all participants provided informed consent. The study population consisted of 45 participants who were scheduled with anthracycline and trastuzumab therapy for HER2-positive early breast cancer and on follow-up at least for 6 months without recurrence. 38 patients had received 90 mg/m^2^ epirubicin for 4 cycles, 5 patients had received epirubicin 100 mg/m^2^ for 6 cycles, and 2 patients had received doxorubicin 50 mg/m^2^ for 6 cycles. Control group included age and cardiovascular risk factors matched 30 adult volunteers, without clinical or documented evidence of cardiovascular or cardiorespiratory diseases who were admitted to internal medicine outpatient clinic. Participants were ineligible for enrolment into both groups if they were older than 75 years, had active infection, had metallic cardiac valve, and were pregnant.

All subjects underwent medical history and a full physical examination. Height and weight, heart rate, and arterial blood pressure measurements were done, and body mass index was calculated for all subjects. Smoking, hypertension, diabetes and medication history, primary characteristics of the tumor, chemotherapy, and radiotherapy protocols were obtained from file records. Fasting blood glucose, lipid profile, and complete blood count which are routinely evaluated in laboratory tests were recorded from file information.

All subjects were submitted to standard echocardiography and functional arterial evaluation by pulse-wave velocity.

### 2.2. Measurements

Ambulatory recording of aortic blood pressure, wave reflections, and arterial stiffness were achieved with the brachial cuff-based oscillometric device Mobil-O-Graph (IEM, Stolberg, Germany) [11]. Common cuff is replaced to the left upper arm. After the conventional oscillometric blood pressure measurement, the cuff reinflates at the diastolic phase for 10 seconds and records brachial pulse waves with a high-fidelity pressure sensor.

### 2.3. Statistical Analysis

Statistical analyses were performed using Statistical Package for Social Sciences for Windows version 23 (SPSS Inc.; Chicago, IL, USA) software package. Shapiro–Wilk test was used to assess all continuous variables for normality prior to data analysis. Continuous variables are expressed as mean ± standard deviation (mean ± SD) or median and range, depending on the distribution. Categorical variables are presented as absolute frequencies and relevant percentages. Comparisons according to the homogeneity of variance were performed by independent sample *t*-test or Mann–Whitney *U* test. Comparison between different groups regarding categorical variables was tested using the Fisher Exact test. Multivariate linear regression analysis was used to evaluate independent variables for PWV. A *p* value less than 0.05 was considered statistically significant.

## 3. Results

### 3.1. Patient Characteristics

This study included 45 patients with the diagnosis of HER2-positive breast cancer who have been exposed to anthracycline- and trastuzumab-based therapy in the adjuvant setting and 30 control volunteers. Demographic characteristics of patient and control groups are shown in Table 1. There was no difference in mean age, body mass index, menopausal status, hypertension, diabetes, and smoking habits between patient and control groups. The control group consisted of 46.7% premenopausal, 10% perimenopausal, and 43.3% postmenopausal women, while breast cancer group included %44.4 premenopausal, %4.4 perimenopausal, and %51.1 postmenopausal women.

### 3.2. Breast Cancer Features and Laboratory Analysis

Breast cancer histological features are shown in Table 2. The most common histological type was invasive ductal carcinoma (91%). The mean tumor size was 2.6 cm. All patients were HER2-positive, and 48.9% of patients had lenfovascular invasion.

Laboratory parameters of patient and control groups were shown in Table 3. The blood glucose values were significantly higher in cancer patients than controls (*p*=0.002). There were no significant differences between HDL cholesterol, LDL cholesterol, triglyceride, hemoglobin, and platelet values. Mean platelet volume was significantly higher in patient group than control group (*p*=0.001).

### 3.3. Treatment Protocols

Treatment protocols of patients are shown in Table 4. Most of the patients (68.9%) had received 4 cycles of EC (epirubicin + cyclophosphamide) followed by paclitaxel and trastuzumab. 88.9% of patients had received radiotherapy and 75.6% of patients had received hormonotherapy.

### 3.4. Arterial Stiffness Measurements

Peripheral and aortic measurement indices by mobilograph are shown in Table 5. Peripheral diastolic blood pressure, central diastolic blood pressure, augmentation index, and pulse wave velocity were significantly higher in patient group than in control group. The mean pulse wave velocity was 7.3 ± 1.2 in patient group and 5.8 ± 1.4 in control group (*p* < 0.01). Pulse wave velocity was not affected by the operation type (*p*=0.5) and existence of radiation treatment (*p*=0.07); however, it was related with the type of hormonal treatment (*p* < 0.01).

Mean PWV was not different between patients taking different chemotherapy regimens. PWV was significantly higher in patients taking aromatase inhibitors than patients receiving tamoxifen (*p* < 0.01). There was no significant difference between patients taking hormonal treatment as compared with patients who did not received hormonal treatment (*p*=0.4). (Table 6).

PWV was significantly higher in patient group than control group both in pre- and postmenopausal setting (*p*=0.02 and *p*=0.02, resp.). There was no significant difference between patient and control group in perimenopausal setting. PWV was significantly higher in postmenopausal patients than pre- and perimenopausal patients (Table 7).

## 4. Discussion

This study demonstrated that breast cancer patients, who received anthracycline- and trastuzumab-based therapy, have an increased aortic stiffness which is a risk factor for future cardiovascular events. Peripheral diastolic blood pressure, central diastolic blood pressure, augmentation index, and pulse wave velocity were significantly higher in breast cancer patients who received anthracycline- and trastuzumab-based chemotherapy. PWV was also significantly higher in patients who were postmenopausal or taking aromatase inhibitors.

Five-year survival rate of breast cancer patients has increased as a result of advances in diagnosis and treatment. However, survival rate of cured patients are still lower than the general population [12]. Although anthracycline-based chemotherapeutic regimens for breast and hematological malignancies have improved cancer-related survival, studies have recognized an unanticipated increase in premature cardiovascular events among individuals receiving these regimens. Breast cancer patients who had five-year disease-free survival after adjuvant therapy have been shown more likely to die due to other than breast cancer-specific reasons.

There is a long period between cancer treatment and symptomatic heart disease. Breast cancer survivors treated with anthracycline-based chemotherapy display cardiac dysfunction 10–15 years after treatment [13]. Therefore, there is a need for noninvasive method that can detect vascular changes before symptoms occur and indicate susceptibility to cardiac events. Aortic stiffness is a risk factor for future cardiovascular events in patients who have not been received cancer therapy, regardless of the Framingham risk score. Arterial stiffness, as an indicator of atherosclerosis, generates a stimulus for left ventricular hypertrophy and triggers subendocardial ischemia by reducing coronary artery perfusion [14].

Currently, cumulative doses of anthracycline-based chemotherapeutic agents are restricted. Epirubicin and doxorubicin are both associated with cardiotoxicity, but epirubicin is less cardiotoxic than doxorubicin. Therefore, epirubicin can be administered at higher cumulative doses (a total of 900 mg/m^2^ for epirubicin versus 450 mg/m^2^ for doxorubicin) [15]. Cumulative dose anthracycline treatment was below the restricted doses for all of our patients. Drafts et al., examined the effect of low- to moderate-dose anthracycline-based chemotherapy in 53 breast cancer, lymphoma, and leukemia patients [16]. Cardiac MRI method is used to measure thoracic aortic pulse wave velocity. PWV significantly increased from baseline to 6 months after initiation of low to moderate doses of anthracycline-based chemotherapy. Anthracycline-based chemotherapy was associated with the early development of subclinical abnormalities of cardiac and vascular function that are associated with the future cardiovascular events.

We used oscillometric technique to determine arterial stiffness. Pulse wave velocity measurement with applanation tonometry or arterial compliance evaluation with ultrasonography or MRI is the other techniques that can be used to determine arterial stiffness.

Although these methods are acceptable, in terms of experimental studies, it is difficult for common use and there is need for more practical methods. Pulse wave analysis obtained from oscillometric techniques are similar with those obtained from tonometric technique. This technique also provides calculation of augmentation index which is an indicator of central arterial stiffness. Feistritzer et al. compared oscillometric device (Mobil-O-Graph) with cardiac magnetic resonance (CMR) to estimate aortic PWV in STEMI patients [17]. They revealed that aortic PWV assessed noninvasively by oscillometric technique showed an acceptable relevance with the CMR-method.

Another method to determine arterial compliance is measuring arterial coupling and circumferential strain with echocardiography. Narayan et al. evaluated breast cancer patients who received doxorubicin and/or trastuzumab therapy to determine chemotherapy-related cardiac dysfunction by using echocardiography-derived measures of arterial elastance. They identified ventricular arterial coupling and circumferential strain as the predictors of chemotherapy-related cardiac dysfunction as a noninvasive strategy to identify high-risk patients [18]. While several techniques exist for detecting arterial stiffness, echocardiographic and oscillometric techniques are very portable, fast, and relatively inexpensive compared to other methods and suitable for general use in clinical practice.

We found that arterial stiffness in postmenopausal women were significantly higher than the pre- and perimenopausal patients. Pulse wave velocity was also significantly higher compared with age- and cardiovascular risk factors-matched control group. Several studies suggest a linear, age-related increase in arterial stiffness. There is controversy about additional effect of menopause independent from age and conventional atherosclerotic risk factors. Zaydun et al. examined 3149 women to determine whether the menopause augments the age-related increase in arterial stiffness [19]. In this study, women who entered menopause at least 6 years previously had the highest PWV independent of age and other atherosclerotic risk factors such as hypertension, hypercholesterolemia, diabetes mellitus, obesity, and smoking. They suggested that the menopause amplified the age-related increase in arterial stiffness during the early postmenopausal phase.

In this study, arterial stiffness was increased in patients receiving aromatase inhibitors compared to patients receiving tamoxifen. Cardiovascular disease has been shown to increase in postmenopausal women in randomized trials. Amir et al. evaluated 30023 postmenopausal women in a meta-analysis to assess life threatening adverse events of adjuvant endocrine therapy and concluded that aromatase inhibitors increased the risk of developing cardiovascular disease compared with tamoxifen endocrine therapy [20]. Cardiovascular risk increase with using aromatase inhibitors may depend on many factors [21]. Aromatase inhibitors have been shown to predispose atherosclerosis by either inducing hypercholesterolemia or direct effect on endothelium [22]. In our study, there was no difference between the cholesterol values in patients using aromatase inhibitors and other patients.

Radiotherapy increases the risk of coronary artery disease. Cardiac toxicity due to radiotherapy is reported before 1980s and decreased with the use of modern techniques. Even though heart is exposed lower doses of radiation with new techniques, some patients with left-sided breast cancer may take toxic doses. These patients have higher risk of developing cardiovascular disease after 10 years. Kilicaslan et al. evaluated 105 HER2-positive breast cancer patients [23]. Patients who received radiotherapy had decreased aortic distensibility as an indicator of arterial stiffness. They concluded that elastic properties are disturbed among patients with breast cancer who received radiotherapy that may contribute increased rate of cardiovascular events. In our study, there was no difference in PWV between patients receiving radiotherapy and other patients. However, patient who did not receive radiotherapy were just 10% of patient population; this may affect the statistical analysis.

In conclusion, arterial stiffening as an indicator of subclinical cardiac damage can be detected with pulse wave analysis which is a reliable, noninvasive, cheap, and practical technique. Routine follow-up of patients receiving antracycline and trastuzumab-based adjuvant regimens may show patients under risk. Further studies are necessary to evaluate whether implementation of cardioprotective strategies such as modification of chemotherapy regimens, or medical intervention strategies could reverse the subclinical changes in patients with increased arterial stiffness.

## Figures and Tables

**Table 1:** Demographic data.

		Control (*n*=30)	Cancer patients (*n*=45)	*p*
Age (years)^∗^		50.3 ± 10.7	53.1 ± 8.6	0.221
Body mass index (kg/m^2^)^∗^		28.2 ± 5.3	30.0 ± 6.0	0.180
Hypertension^∗∗^	No	27 (90)	39 (86.7)	0.733
	Yes	3 (10)	6 (13.3)	
Smoking^∗∗^	No	27 (90)	42 (92.3)	0.229
	Yes	3 (10)	3 (6.7)	
Diabetes^∗∗^	No	26 (86.7)	39 (86.7)	1.000
	Yes	4 (13.3)	6 (13.3)	
Menopausal status^∗∗^	Premenopausal	14 (46.7)	20 (44.4)	0.583
	Perimenopausal	3 (10)	2 (4.4)	
	Postmenopausal	13 (43.3)	23 (51.1)	

^∗^Mean ± standard deviation.^∗∗^Frequency (percent).

**Table 2:** Breast cancer characteristics.

		*n* (%)
Tumor diameter (median)		2.5 (0.5–5)
Histological type	Invasive ductal	41 (%91)
	Micropapillary	1 (%2.2)
	Apocrine	1 (%2.2)
	Medullary	1 (%2.2)
	Inflammatory	1 (%2.2)
Lenfovascular invasion	Yes	18 (%48.9)
	No	22 (%40)
	Unknown	5 (%11.1)
Grade	Grade 1	3 (%6.7)
	Grade 2	23 (%51.1)
	Grade 3	6 (%13.3)
	Unknown	13 (%28.9)
ER	Positive	30 (%66.7)
	Negative	15 (%33.3)
PR	Positive	29 (%64.4)
	Negative	16 (%35.6)
HER 2	Positive	45 (%100)
	Negative	0

ER: estrogen receptor; PgR: progesteron receptor; HER2: human epidermal growth factor receptor.

**Table 3:** Laboratory parameters.

	Control (*n*=30)	Patients (*n*=45)	*p*
Blood glucose^∗∗^	90 (67–144)	97 (73–242)	**0.002**
LDL^∗^	120.2 ± 25.1	142.1 ± 33.4	0.06
HDL^∗^	49.9 ± 11.4	48.8 ± 11	0.728
Triglyceride	111.5 (50–331)	122.5 (65–321)	0.102
Cholesterol	195.6 ± 32.8	220.9 ± 38.8	**0.009**
Hemoglobin	13 (10.7–17.6)	12.9 (9.4–14.9)	0.397
Hematocrit	39.8 ± 4.2	38.7 ± 3.1	0.226
PCT^∗∗^	0.3 (0.2–3.9)	2.3 (0.2–3.6)	**<0.001**
Mean platelet volume	8.8 ± 1.3	9.8 ± 1.2	**0.001**

LDL: low density lipoprotein; HDL: high density lipoprotein; PCT: platelet crit.

**Table 4:** Treatment characteristics.

		*n* (%)
Operation^∗∗^	Modified radical mastectomy	19 (42.2)
	Breast conserving surgery	26 (57.8)
Chemotherapy^∗∗^	Anthracycline + taxane + trastuzumab	34 (75.6)
	Anthracycline + trastuzumab	10 (22.2)
	Taxane + trastuzumab	1 (2.2)
Radiotherapy	No	5 (11.1)
	Yes	40 (88.9)
Hormonotherapy^∗∗^	No	11 (24.4)
	Yes	34 (75.6)
Hormonotherapy drug	Tamoxifen	17 (50)
	Aromatase inhibitors	17 (50)

**Table 5:** Mobilograph measurement indices.

	Control (*n*=30)	Patient (*n*=45)	*p*
pSBP (mmHg)^∗^	116.7 ± 16.1	123.3 ± 11.9	0.050
pDBP (mmHg)^∗^	73.4 ± 14	83 ± 10.6	**0.001**
pMAP (mmHg)^∗^	92.3 ± 13.2	102.2 ± 12	**<0.001**
bPP (mmHg)	42.1 ± 13.9	43.5 ± 10.7	0.66
cSBP (mmHg)^∗^	108.6 ± 16.3	114.9 ± 15	0.090
cDBP (mmHg)	74.8 ± 14	84.5 ± 10.6	**0.001**
aPP (mmHg)^∗^	33.8 ± 9.1	30.4 ± 10.1	0.144
CO (L/min)^∗^	4.1 ± 0.5	4.4 ± 0.7	0.077
Peripheral resistance^∗^	1.2 ± 0.1	1.3 ± 0.2	0.160
CI (L/m^2^ × 1/m^2^)^∗∗^	2.2 (1.7 to 3)	2.4 (2 to 5.6)	0.074
Augmentation pressure^∗^	7.4 ± 6	7.3 ± 4.1	0.868
Augmentation index^∗∗^	23.5 (−19 to 42)	29.5 (1 to 53)	**0.010**
CO^∗∗^	4 (3.2 to 5.5)	4.4 (3.3 to 8.3)	0.050
Pulse wave velocity^∗^	5.8 ± 1.4	7.3 ± 1.2	**<0.001**

pSBP: peripheral systolic blood pressure; pDBP: peripheral diastolic blood pressure; pMAP: periferic mean arterial pressure; bPP: brachial pulse pressure; cDBP: central diastolic blood pressure; cSBP: central systolic blood pressure; aPP: aortic pulse pressure; CO: cardiac output; CI: cardiac index.

**Table 6:** PWV according to treatment protocols.

	Mean ± SD	*p*
Operation	Lumpectomy (*n*=26)	7.2 ± 1.2	0.503
	Modified radical mastectomy (*n*=19)	7.4 ± 1.3	
Radiotherapy	Yes (*n*=40)	7.2 ± 1.2	0.076
	No (*n*=5)	8.2 ± 1.0	
Chemotherapy	Anthracycline + trastuzumab	7.6 ± 1.0	0.428
	Anthracycline + taxane + trastuzumab	7.2 ± 1.3	
Hormonotherapy	Yes (*n*=34)	7.4 ± 1.2	0.434
	No (*n*=11)	7.0 ± 1.4	
Hormonotherapy type	Tamoxifen (*n*=17)	6.5 ± 0.7	<0.01
	Aromatase inhibitor (*n*=17)	8.3 ± 0.9	

EC: epirubicin + cyclophosphamide; FEC: fluorouracil + Epirubicin + Cyclophosphamide.

**Table 7:** Menopausal Status and pulse wave velocity.

	Control (*n*=30)	Patient (*n*=45)	*p*
Premenopausal	5.2 ± 1.2	6.4 ± 0.8	0.002
Perimenopausal	5.2 ± 1.1	6.0 ± 0.7	0.424
Postmenopausal	6.5 ± 1.5	8.2 ± 0.8	0.002
